# Overcoming the Odds: Successful Treatment of Disseminated Mucormycosis with Gastrointestinal and Jaw Involvement in a Patient with Acute Myeloid Leukemia

**DOI:** 10.1155/2023/5556540

**Published:** 2023-10-03

**Authors:** Thuraya Al-Busaidi, Fatma Al-Bulushi, Adil Al-Zadjali, Abdulaziz Bakathir, Abdullah Balkhair, Ibrahim Al Busaidi

**Affiliations:** ^1^Department of Hematology, Sultan Qaboos University Hospital, SQU, Muscat, Oman; ^2^Department of General Surgery, Sultan Qaboos University Hospital, SQU, Muscat, Oman; ^3^Department of Dental and Maxillofacial Surgery, Sultan Qaboos University Hospital, SQU, Muscat, Oman; ^4^Infectious Diseases Unit, Department of Medicine, Sultan Qaboos University Hospital, SQU, Muscat, Oman

## Abstract

Disseminated mucormycosis is a rare life-threatening fungal infection that is uniquely seen in severely immunocompromised patients including those with hematological malignancies. We report a case of disseminated mucormycosis with a biopsy-proven gastrointestinal and oral cavity involvement in a patient with acute myeloid leukemia during induction chemotherapy. The patient had a successful outcome with limited resection of the involvement bowel segment, multiple maxillary dental extractions, debridement of the alveolus and hard palate, and combined antifungal therapy. After clinical improvement, stable infection on serial abdominal imaging, and completion of 6 weeks of combined antifungal therapy, consolidation chemotherapy was given, and molecular remission was achieved. The patient remained clinically well on secondary antifungal prophylaxis.

## 1. Introduction

Mucormycosis is a fungal infection generally caused by filamentous molds belonging to the orders Mucorales and Entomophthorales. This aggressively serious infection is largely seen in immunocompromised patients. These include patients with poorly controlled diabetes mellitus, solid organ transplant recipients, patients with hematological malignancies, and recipients of allogeneic stem cell transplants [1–3].

The use of systemic corticosteroids is another risk factor for invasive mucormycosis, which has been more recently observed in patients with COVID-19 infection [2, 4, 5].

Invasive mucormycosis is characterized by an overwhelmingly high mortality rate that varies depending on the affected sites. According to the largest available retrospective study, which included 929 reported cases of zygomycosis, the mortality rate was approximately 96% for disseminated disease and 85% for patients with gastrointestinal (GI) mucormycosis [2].

Allogeneic stem cell transplant recipients have the highest incidence rate for mucormycosis with an estimated incidence rate of 1.19% followed by acute lymphoblastic leukemia at 0.75%, while patients with acute myeloid leukemia had an estimated incidence rate of 0.45% [6].

Severe graft versus host disease, high-dose corticosteroids, previous CMV infection, and increasing age are the main risk factors [6, 7] Rhino-orbital-cerebral mucormycosis is the most common form of the disease, however, in patients with hematological malignancies and allogeneic stem cell transplant recipients, pulmonary mucormycosis is most common form followed by rhino-orbital-cerebral mucormycosis (ROCM) and disseminated mucormycosis [2, 4, 6, 8, 9].

## 2. Case Presentation

A previously healthy 17-year-old girl was admitted to the Sultan Qaboos University Hospital (SQUH) on January 12th, 2023, for evaluation of significantly elevated white cell counts. She initially presented to a local hospital with symptoms of an upper respiratory tract infection. Routine laboratory investigations revealed an elevated white cell count, anemia, and thrombocytopenia. A peripheral blood smear showed circulating blasts, and a bone marrow biopsy confirmed the diagnosis of acute myeloid leukemia (monocytic) with 67% blasts on bone marrow aspirate. Cytogenetic and molecular testing revealed inversion 16 (inv16). Upon admission, she exhibited clinical evidence of gingival infiltration with leukemia. Cultures were collected and piperacillin-tazobactam was initiated empirically for febrile neutropenia. She commenced oral posaconazole for antifungal prophylaxis along with other antimicrobial prophylaxis routinely used during the induction period.

The patient required cytoreduction with hydroxyurea before commencing induction chemotherapy with cytarabine and daunorubicin. On day +3 of induction, she developed persistent high-grade fever, leading to escalating antibiotics cover to meropenem and vancomycin intravenously. Blood cultures collected from the central venous catheter and peripheral vein were sterile. Cytarabine was interrupted for 48 hours for suspected cytarabine syndrome as all infection workup and cultures were negative. Chemotherapy was resumed with hydrocortisone premedication once daily given 30 minutes preinfusion. The fever subsided, and all induction chemotherapy infusions were completed.

**Figure 1:**
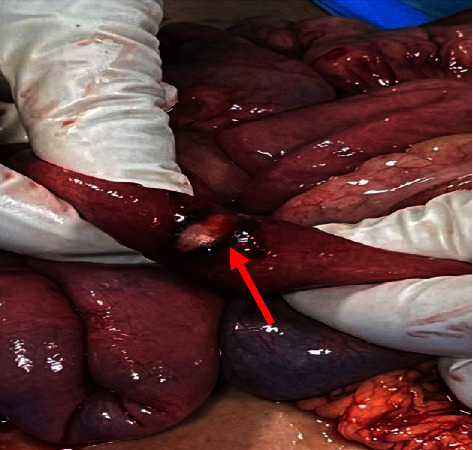
Intraoperative findings of the small bowel. The picture shows a 2 cm necrotic segment in the ileum, located 40 cm from the ileocecal valve.

On day +16 of induction, she developed acute abdominal pain. An urgent abdominal computerized tomography (CT) scan revealed significant thickening of the cecum, ascending colon, and splenic flexure, along with mural thickening affecting the terminal ileum and sigmoid. There were also areas of wall thinning in multiple segments of the colon, raising concerns about transmural necrosis. In consultation with the general surgery team, she underwent an emergency exploratory laparotomy. Intraoperatively, the bowel appeared normal, except for a 2 cm necrotic segment in the ileum, located 40 cm from the ileocecal valve (as shown in Figure 1). Approximately 8 cm of the ileum was resected, and an end ileostomy was created.

**Figure 2:**
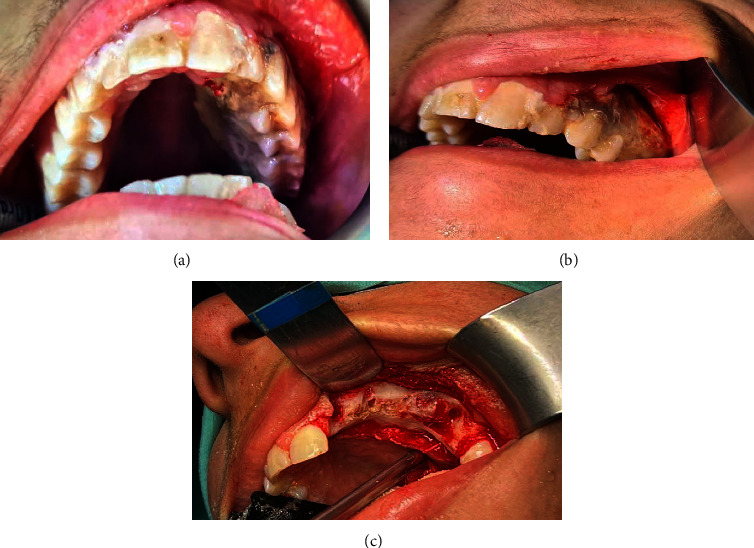
(a, b) Preoperative images showing extensive gingival and palatal necrosis extending from the maxillary left lateral incisor to the molar region. (c) Intraoperative image showing avascular and maxillary necrotic alveolar and palatal bone.

**Figure 3:**
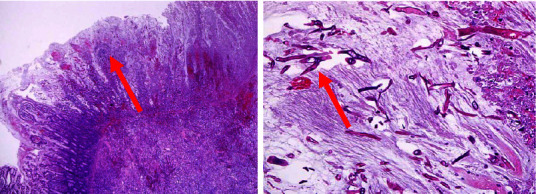
Terminal ileum biopsy showing fungal hyphae.

Postoperatively, the patient continued to have nonremitting high-grade fever and constant abdominal pain. The patient was initiated on combination antifungal therapy, consisting of liposomal amphotericin B at a dosage of 10 mg per kg per day and caspofungin at 70 mg loading dose and then 50 mg daily, and continued on oral posaconazole suspension of 200 mg every 8 hours. The granulocyte colony-stimulating factor (GCSF) was also started to expedite neutrophil recovery. Furthermore, the patient developed a painful lesion at the hard palate which progressed to a whitish necrotic area with dusky margins (Figure 2). Scrapings from the lesion were sent for fungal culture. Subsequently, *Rhizopus* species were identified from these scrapings confirming the diagnosis of oral mucormycosis. In addition, *Candida orthopsilosis* was also isolated from the same culture. Histopathological examination of the resected ileum segment revealed extensive mucosal ulceration, transmural necrosis, and hemorrhages. Numerous broad, ribbon-like, pauci-septate fungal hyphae with irregular walls and branching randomly at various angles were observed (Figure 3). In addition, extensive angioinvasion was also noted. These histopathological findings were consistent with invasive intestinal mucormycosis. Similar findings of necrosis and infiltration with a dense acute inflammatory cell containing aggregates of fungal hyphae were also observed in the omental biopsy. A diagnosis of disseminated invasive mucormycosis with the involvement of two noncontiguous sites was made.

A multidisciplinary team approach involving infectious diseases, general surgery, maxillofacial surgery, and primary leukemia teams concluded that debridement and debulking of the alveolus, hard palate, and surgical resection of the involved bowel is paramount to the management of this condition. Nevertheless, in view of the patient's immunocompromised state, the multifocal disseminated nature of the disease, and the inherently associated and largely prohibitive surgical and postsurgical complications, a closely observant, conservative approach of the abdominal pathology with optimization of antifungal therapy and reversion of immune suppression state was contemplated in consultation with the patient and patient family. The patient underwent debridement of the hard palate and extraction of the involved teeth. Histopathological examination of the resected bone and necrotic tissue from the hard palate revealed mucormycosis. The patient made remarkable clinical improvement with abatement of fever, regression of the palatal lesion, and resolution of the abdominal pain.

Combined antifungals were continued. A follow-up abdominal CT six weeks postoperatively while on combination antifungal therapy demonstrated stable disease with residual colonic wall thickening. During this time, the patient continued to improve and was symptom-free. An additional six weeks of combination of high-dose liposomal amphotericin B and caspofungin was given.

From acute myeloid leukemia perspective, the patient achieved morphological complete remission by the end of induction. Following this, she received a bridging period of four weeks with azacitidine to high-dose cytarabine without any complications. She tolerated high-dose cytarabine (HIDAC) consolidation and achieved molecular remission following the first consolidation. A follow-up CT abdomen during consolidation was performed which showed minimal residual changes three months later.

## 3. Discussion

We described a successful outcome of a case of disseminated mucormycosis with gastrointestinal and jaw involvement complicating induction chemotherapy in a patient with acute myeloid leukemia.

Among all cases of mucormycosis, gastrointestinal (GI) mucormycosis constitutes approximately 4–7% of all cases [2, 8]. The stomach is the most frequently affected site within the gastrointestinal tract, although other sites such as the ileum, colon, and liver can also be involved [2, 10]. It is acquired by ingestion of pathogens or spores in food products. In comparison with solid organ transplant recipients, the incidence of intestinal mucormycosis in patients with hematological malignancies is more common than in gastric form due to increased frequency of neutropenia, mucositis, and neutropenic enterocolitis which lead to the breakdown of mucosa and invasive fungal infection [9, 10]. The clinical presentation of GI mucormycosis varies depending on the affected site. Most patients present with nonspecific symptoms indicative of acute abdomen, such as abdominal pain, nausea, and vomiting [9, 11]. GI bleeding was reported in 48% of patients in one study [9]. Symptoms can progress rapidly with imminent risk of bowel perforation [11] Immunocompromised patients may also experience persistent high-grade fever that does not respond to broad-spectrum antibiotics. Our patient developed high-grade nonremitting fevers in addition to acute abdominal pain with concerns for bowel ischemia on the CT abdomen which triggered an urgent surgical intervention.

Diagnosing GI mucormycosis is usually challenging, and only 25% of cases are diagnosed antemortem, with only a few cases diagnosed based on the pathology of resected bowel following surgical intervention [5, 12]. The pathological hallmark of GI mucormycosis is tissue infarction resulting from hyphae angioinvasion [11, 13]. The characteristic histopathologic features are the presence of aseptate wide hyphae with focal bulbous and nondichotomous branching, occasionally at right angles. Growth of Mucorales from the resected tissue was reported only in 30% of surgical samples. Abdominal imaging findings are nonspecific including the presence of mass, thickening of the wall, and signs of bowel perforation [13].

Our patient has significant thickening of the colon and thinning of some segments suggestive of bowel necrosis. This lethal infection should be suspected in neutropenic patients with persistent fever and severe abdominal pain as it is illustrated in our case.

Early recognition and diagnosis are the key factors for successful treatment of gastrointestinal (GI) mucormycosis. Surgical debridement and debulking of the affected tissues are paramount in the control of infection in combination with antifungal therapy [11, 14]. High-dose (7.5–10 mg/kg per day) liposomal amphotericin B is the antifungal of choice. Isavuconazole is a broad-spectrum azole that was shown to have comparable efficacy to liposomal amphotericin B in the treatment of mucormycosis and it can be considered as an alternative therapy in patients who have contraindications or intolerance to high-dose liposomal amphotericin [11, 14, 15]. The addition of an echinocandin to liposomal amphotericin B may potentially improve outcomes, although robust evidence is lacking. Antifungal therapy alone is generally insufficient for this pathology due to poor penetration of antifungal agents into the affected bowel parts by angioinvasion of the mold and compromised blood supply [11, 14]. Our patient received combined antifungal therapy with high-dose liposomal amphotericin and caspofungin in addition to oral posaconazole to provide a local antifungal effect in the involved bowel.

Antifungal therapy should be continued for a minimum of 6 weeks, and if possible, immunosuppressive medications should be avoided during this period. The optimal duration of antifungal treatment beyond 6 weeks is not well-established and it depends on multiple factors including the resolution of clinical symptoms and signs, resolution of the immune defects, and radiological response to therapy [11, 14]. Most patients will receive secondary prophylaxis for up to one year following the initial therapy, based on case reports and expert opinion.

Our patient received high-dose liposomal amphotericin B in combination with caspofungin and posaconazole for a duration of six weeks. Posaconazole was subsequently stopped after 6 weeks of treatment, and she remained on dual antifungal therapy for an additional 6 weeks. In view of her clinical improvement and stable disease on imaging, caspofungin was stopped after 12 weeks of combined antifungal therapy. The patient was maintained on high-dose amphotericin B in view of anticipated ongoing immunosuppression with consolidation chemotherapy. Serial abdominal imaging showed persistent bowel thickening. The standard of care consolidation was not feasible following induction given her active GI mucormycosis. Given her improved clinical status and stable radiological, she was commenced consolidation with azacitidine at a dose of 75 mg/m^2^ daily for 7 days during the first cycle, followed by high-dose cytarabine at 3 g/m^2^ intravenously twice daily on days 1, 3, and 5 during the second cycle of consolidation. There are no available guidelines to address the management of chemotherapy in patients with hematological malignancies during active fungal infection. Our patient is considered for secondary prophylaxis with oral isavuconazole and until it becomes available, she will be maintained for three days per week on liposomal amphotericin B for the remaining duration of her acute myeloid leukemia (AML) treatment.

## 4. Limitations

While our case report provides valuable insights into the management and successful outcome of disseminated mucormycosis in a severely immunocompromised patient, it is essential to acknowledge certain limitations in our study. The histopathological and microbiological confirmation of mucormycosis in our case is robust, establishing it as the primary pathology. However, it is crucial to recognize that such definitive confirmation may not always be feasible when a surgical approach is appropriately not taken for colitis in this unique population. Neutropenic enterocolitis, for instance, is a leading cause of acute abdomen mucormycosis in such patients, which generally does not warrant immediate surgical intervention in most cases.

Furthermore, we cannot definitively establish a causal relationship between the treatment our patient received and her overall outcome, as our findings are based on a single case.

## 5. Conclusion

Our case highlights multiple learning points including the importance of early clinical recognition, histopathological diagnosis, and surgical management in combination with high-dose liposomal amphotericin B in successful management of disseminated mucormycosis in patients with hematological malignancies. It also highlights that the initiation and resumption of chemotherapy during active fungal infection is challenging and it has to be individualized.
